# Cultural adaptation and psychometric adequacy of the Persian version of the physical activity scale for the elderly (P-PASE)

**DOI:** 10.1186/s13104-019-4591-7

**Published:** 2019-09-02

**Authors:** Leila Keikavoosi-Arani, Leili Salehi

**Affiliations:** 1 Department of Health Services Management, School of Health, Research Center for Health, Safety and Environment, Alborz University of Medical Sciences, Karaj, Iran; 2 Research Center for Health, Safety and Environment, Health Education and Promotion, Alborz University of Medical Sciences, Karaj, Iran

**Keywords:** Physical activity for the elderly, PASE, Validity, Reliability, Psychometric adequacy

## Abstract

**Objective:**

This cross sectional study was conducted to investigate cultural adaption and validation of the Persian version of the PASE among the elderly community dwellers in Iran. Out of 278 elderly people, 65% of them were female. After translation and cultural adaption, the PASE was evaluated with respect to the validity and reliability. Regarding the construct validity, the concurrent validity was assessed between the PASE and ADL, IADL, self-rated health, and TUG test.

**Results:**

The mean score of P-PASE was equal to 153.73 ± 48.47. P-PASE scores were significantly and negatively correlated with TUG (r = − 0.691, P < 0.001) and age (r = − 791, P > 0.001), and were also significantly correlated with ADL (r = 0.775, P < 0.001), and IADL (r = 0.161, P < 0.001). The ICC was obtained as 0.92 (95% CI 0.90–0.94), 0.86 (95% CI 0.82–0.87), and 0.91 (95% CI 0.90–0.94) for the leisure time activity, household activity, and work-related activity scores, respectively. The Cronbachs̓ alpha coefficient was equal to 0.74, 0.74, and 0.79, respectively for leisure time activity, household activity, and work-related activity domains.

## Introduction

Physical activity (PA) has a critical role in the prevention of diseases, increasing the level of independency and improving the quality of life [1]. Despite the known benefits of PA, physical inactivity has remained as a global concern [2].

The analysis of physical inactivity levels among different age groups showed that, the prevalence of physical inactivity is higher among elderly. In line with the concern to promote PA among the population, researchers have been highly interested in measurement of PA so far. Questionnaires are instruments normally used in epidemiological studies mainly providing the opportunity for assessment of PA in a large sample size [3].

Previous studies have reported the measurement properties of several ‘past-day’ and ‘last-week’ recall methods [4–6]. Several questionnaires have been developed in this regards. Among these, the physical activity scale for elderly (PASE) [7] had more advantages compared to the others as short practice period, the easy scoring process, and its applicability via letters or phone. Separately, it consists of 3 subheadings including leisure time (5 components), household (4 components), and work -related activities (1 component). Walking outside, light/moderate and strenuous sports and activities focusing on muscle strength are categorized as leisure time. Household activities consist of light/heavy housework, home repairs, lawn work/yard care, outdoor gardening, and caring for another person. Work-related activity subheading compromises of jobs involving standing [7].

This instrument has been translated and validated in various cultures [8–10]. But despite the importance and ease of use, cultural adaption and validation of the PASE have not been investigated so far in Iran. This study was designed to investigate the cultural adaption and validation of the PASE among the elderly in Iran.

## Main text

### Methods

#### Design

This cross sectional study was conducted in four phases. The first and second phases included the translation of the scale and modifying it, the third phase included the evaluation of the content and face validity, and during the fourth phase the construct validity and reliability were assessed.

### Phase 1

#### Translation

After obtaining permission from Washburn first developers of PASE [7], at the first, the original (English) version of the questionnaire was translated to Persian by two independent translators, and then this Persian version was translated to the original language by another two independent translators. Finally, the two versions were compared.

### Phase 2

#### Modified version

Two expert panels (each one consisted of 5 geriatric specialists) reviewed the PASE, altered some activities with respect to the Persian culture, and added some items. The intensity of the replaced activities was determined by the expert’s panels (Table 1).

**Table 1:** The modified items were summarized

PASE activities	Replaced activities in the Persian version
Walking the dog	Walking with your friends or neighborhood
Bowling, golf with a cart, shuffleboard, fishing from a boat or pier	Playing backgammon or chess, go shopping just to take a break
Doubles tennis, ballroom dancing, hunting, ice skating, golf without a cart, softball	Participation and giving services in religious ceremonies or going marches with family and friends
Gagging, swimming, cycling, singles tennis, aerobic dance, skiing (downhill or cross-country)	Playing with sports equipment in the parks, mountain climbing
Carrying the wood	Washing and repairing the car
Seating assembly line worker	Rosary
Walking along with handling some materials generally weighing less than 50 lbs (including mailman, waiter/waitress, construction worker, heavy tool and machinery worker)	Buying fruits and vegetables and other groceries (by kilograms)
Lumberjack, stonemason, farm or general laborers)	Carrying house furniture during moving to a new house, farming and laboring

Furthermore, ironing and washing clothes by hand were added to the heavy housework activities.

### Phase 3

#### Pre-final version

Content and face validity was assessed. For assessing the content validity, content validity ratio (CVR) and content validity index (CVI) were computed. In calculation of the CVR, the Lawshe’s table was used to compare the results. All items were kept in the scale due to obtaining acceptable level (0.62). During CVI computation, all items were also maintained in the scale due to obtaining a CVI > 0.79. For assessing the face validity, the views of 20 Iranian elderly were considered and some items were changed due to their opinions.

### Phase 4

#### Participations

The study population consisted of all the elderly aged 60 years old and above with no cognitive impairment (Mini-Mental State Examination score ≥ 24). The elderly with impaired or surgical limited activity were excluded. The sample size consisted of 278 elderly aged 60–89 years old. The rule of thumb, such as 5 or 10 participants per item was applied for the sample size calculation [11]. Subjects were selected using convenience sampling method.

The main investigator (LS) administered the survey questionnaire and was available to answer the possible questions. 45 min of time was considered to complete the questionnaires.

#### Outcome measurements

I. Physical activity scale for the elderly (PASE) included 12 items asking about the activities performed during 7 days ago. Weight, frequency and duration of each activity were assessed in this questionnaire. The total PASE score was computed by multiplying the amount of time spent in each activity (h/day) or activity participation (yes/no) by the weights of the items obtained and then, summing the results [7].
II. Activities of daily living (ADL) refer to people's daily self-care activities. Health professionals often consider a person's ability or inability to perform daily living activities as a measurement of their functional status, particularly in regard to people with post-injury complications, disabilities and the elderly. ADL scales included feeding, bowel and bladder control, dressing and undressing, chair/bed transferring, bathing self, and toileting [12]. Independence in the given function received 1 point while in case of dependency, 0 point was given.
III. Instrumental activities of daily living (IADL) measure included 7 items: (use of telephone, shopping, food preparation, doing housework, ability to handle finances, responsibility for own medication, and transporting outdoors). IADLs are a basis for assessing participants’ difficulty with (I) ADL. IADLs are those activities whose accomplishment is necessary for continued independent residence in the community. The data were coded as 0, 1, and 2, respectively in case of dependency, needing someone’s help, and independency; so that higher scores indicate higher dependency. Both scales of ADL and IADL were validated in the Persian culture [13]
IV. Self-rated health (SRH) scale measured peoples̓ SHR status, ranging from excellent to poor health status. The question was ‘How would you rate your health today?’ with the following: Excellent; Good; Fair, and Poor.
V. The timed up and go (TUG) test was performed for each participant considering distance in meters and time in seconds. The elderly was asked to stand up from the chair, and walk for 3 meters and then, turn again and walk back toward the chair and sit on it [14, 15]. TUG has been shown to be reliable and valid in measuring the balance, functional mobility, and fall risk in older adults.
VI. Other variables studied in the current paper were: age, sex, educational level, height (cm), weight (kg), and body mass index (BMI).

### Statistical analysis

Data normality was assessed using KS. Descriptive data were presented as M ± SD, while validity and reproducibility results were presented with the corresponding 95% CI. Differences between men and women were investigated using Independent t-test. ICC and Cronbach’s α coefficient were applied to study the stability and internal consistency, respectively by SPSS ver 19. Acceptable level of ICC for the sample size more than 50 was equal and more than 0.7 [16].

To compute ICC, we gave the same test twice to the same people at 2 weeks' interval and then the correlation between the two scores were determined.

#### Concurrent validity

To assess the construct validity, it is recommended to use comparison instruments measuring similar constructs with adequate measurement properties [16], therefore in the present study, the concurrent validity assessment was considered for assessment of construct validity. In this regard, the concurrent validity was assessed between PASE and ADL (I) ADL, SRH, and TUG test.

### Ethical consideration

This study was approved by the Ethics Committee of Alborz University of Medical Sciences (Code no. 1396.207). An informed written consent was obtained from all the participants after explaining the aims and methods of the study. The elderly could withdraw from the study any time before or during the study.

### Results

#### Demographic characteristics

The mean age of participants was equal to 74.22 ± 14.8 years old, 65% of participants were female, and all of them were literate. Other characteristics are presented in Table 2.

**Table 2:** The demographic characteristics and outcome measures results

	Participants (n = 287)			
	Mean	SD	Min	Max
Age (mean ± SD)	74.22	14.8	60	89
Height (cm)	157.69	6.72	142	173
Weight (kg)	70.06	9.86	39	97
BMI	28.24	9.86	18.50	40.16
PASE	153.57	48.47	3	315.74
ADL	15.97	0.47	8	16
IADL	11.07	1.99	0	14
TUG test	1.99	0.89	0.2	9

*BMI* body mass index, *PASE* physical activity scale for the elderly, *ADL* activity daily living, *IADL* instrumental activity daily living, *TUG test* the timed up and go test

The mean P-PASE was equal to 153.73 ± 48.47. The household activities contributed to 53% of the P-PASE mean score (Table 3). The men and female characteristics compared in Additional file 1: Table S1.

**Table 3:** The mean of P-PASE components, ICC and Cronbach’s alpha coefficients for each component

PASE component	Sample mean	Weight	Contribution to total PASE score	ICC (95% CI)	Cronbach’s alpha
Leisure time activity (M ± SD)	48.38 ± 27.85			0.92 (0.90–0.94)	0.74
Walking (h/day)	1.99	20	39.8	0.90 (0.92–0.94)	0.74
Light sport (h/day)	0.14	21	2.94	0.89 (0.87–0.91)	0.71
Moderate (h/day)	0.09	23	2.07	0.93 (0.90–0.95)	0.79
Strenuous sport	0.14	23	3.22	0.91 (0.89–0.92)	0.74
Muscular Strength/endurance (h/day)	0.01	30	0.3	0.92 (0.90–0.95)	0.70
House hold activity (M ± SD)	81.13 ± 33.14			0.86 (0.82–0.87)	0.74
Light housework (%)	88.51	25	22.13	0.86 (0.82–0.86)	0.70
Heavy housework (%)	85.35	25	21.34	0.81 (0.80–0.84)	0.75
Home repair (%)	19.51	30	5.85	0.76 (0.72–0.77)	0.72
Lawn work/yard care (%)	34.49	36	12.42	0.80 (0.79–0.81)	0.75
Caring for another person (%)	55.40	35	19.39	0.95 (0.92–0.97)	0.76
Work for pay or volunteer	24.22 ± 10.83				
Job-standing or walking (h/day)	1.15	21	30	0.91 (0.90–0.94)	0.79
Total PASE score (M ± SD)	153.73 ± 48.47				

#### Concurrent validity

A significant correlation was found between the score of the P-PASE and ADL (r = 0.775, P < 0.001) and also IADL (r = 0.161, P < 0.001). There was a significant negative correlation between the P-PASE score and TUG (r = − 0.691, P < 0.001) and age (r = − 0.791, P < 0.001). No correlation was found between P-PASE and BMI.

#### Reliability

The ICC values of the P-PASE components varied between 0.76 and 0.93 (good to excellent). The internal consistency of the P-PASE components was good, ranged from 0.70–0.79 (Table 3).

### Discussion

The results of the present study showed that, the mean P-PASE score was higher than that obtained in the previous studies [17–19]. According to the developing country’s protocol [20], the age of 60 years old was considered as the onset of ageing; therefore, in the present study, participants̓ age was equal to 60 years old or more, while other studies have been conducted on the individuals aged over 65 years old. Seemingly, the higher P-PASE score in our study was related to younger age of participants. Furthermore, this result is supported by our results obtained regarding the significant negative correlation between the mean of P-PASE score and the age.

In line with other studies [21, 22], our study showed that the females were more physically active than male. Some studies suggested that, the males had more PA than females [23]. The studies conducted by Shuite [21] and KU [24] showed that, the higher PASE score in women is related to the socio-cultural factors. Indeed, in the context of Iranian living, elderly women have high levels of physical activity due to their daily duties such as household activities, caring for grandchildren and so on [22, 25]. Due to current study results, care for another person accounted for 24% of household-related activities. Contribution of the household activities estimated for 53% of the mean P-PASE score, attributing to the higher number of women (65.2%) than men (34.8%). The present finding is consistent with those of other studies [26, 27]. Clinicians should be noticed that sedentary lifestyle in older men could lead to loneliness, isolation, poor health, mobility limitations and depressive symptoms [28].

Similarly, in a study by Dinger [29], contribution of the work-related activities was reported nearly 16% of the mean P-PASE score which is different from other studies [1, 27]. This difference may be due to the socio-cultural differences between Iran and other countries. In Iran, after retirement, the elderly are responsible for doing works for family members such as shopping, going to bank, and various offices to do works related to family members or having second job due to low pension. Therefore, as predicted the work-related activities subheading level of the individuals was higher in our study group compared to other studies.

On the other hand, similar to the study by AYAT [1], the score of leisure time is higher than that obtained in other studies [18, 26]. The findings of this study showed that, walking accounted for 82.26% of leisure time activities and like Turkey, in Iran, [1] walking is an option of activity during leisure time.

In this study, a significant strong correlation was found between ADL and PASE scores. Also, there was a significant correlation between PASE and I (ADL). Other studies suggested that, there is a significant relationship between PASE and IADL. In a study conducted by Ismael [27], a weak to moderate correlation was found between the PASE and the functional performance. The mean PASE had a significant positive correlation with IADL, gait speed, and power of gait.

In the present study, there was a significant strong negative correlation between TUG and the P-PASE score. Similar results were found by other studies [17, 18]. The time spent on completion of TUG has been shown to be linked to PA [30].

Along with the original version [7], there was no correlation between PASE and BMI in the current study, but there was a significant correlation between PASE and health status. Another study showed that, the higher PASE score is correlated with more favorable health [31, 32]. In the current study, the Cronbach's α coefficient indicating the good internal consistency. Other studies have calculated the total Cronbachs̓ α value [33], but it was not computed in our study, the calculating is not necessary for the whole tool, in a study on the instruments composed of different parts [34]

### Conclusion

The results showed that, P-PASE (Additional file 2) is a reliable and valid instrument to assess PA among the elderly in Iran.

## Limitations

Recalling bias among elderly was considered as a limitation.

## Supplementary information

**Additional file 1: Table S1.** The mean of P-PASE components in the male and female.

**Additional file 2.** Persian version of PASE.

## Data Availability

All datasets in this study are available in reasonable request.

## Abbreviations

PASE: physical activity scale for the elderly
P-PASE: Persian version of the physical activity scale for the elderly
PA: physical activity
ADL: activities of daily living
IADL: instrumental activities of daily living
TUG: the timed up and go test
SRH: self-rated health
ICC: intra class correlation coefficient
KS test: Kolmogorov–Smirnov test

## References

[1] Ayavat E, Kilinç M, Kirdi N (2017). The Turkish version of the physical activity scale for the elderly (PASE): its cultural adaptation, validation, and reliability. Turk J Med Sci.

[2] Dumith SC, Hallal PC, Reis RS, Kohl HW (2011). Worldwide prevalence of physical inactivity and its association with human development index in 76 countries. Prev Med.

[3] Ueno DT, Sebastiao E, Corazza DI, Gobbi S (2013). Methods for assessing physical activity: a systematic review focused on older adults. Rev Bras Cineantropom Desempenho Hum.

[4] Olds TS, Ridley K, Dollman J, Maher CA (2010). The validity of a computerized use of time recall, the multimedia activity recall for children and adolescents. Pediatr Exerc Sci.

[5] Matthews CE, Keadle SK, Sampson J, Lyden K, Bowles HR, Moore SC, Libertine A, Freedson PS, Fowke JH (2013). Validation of a previous-day recall measure of active and sedentary behaviors. Med Sci Sports Exerc.

[6] Clark BK, Winkler E, Healy GN, Gardiner PG, Dunstan DW, Owen N, Reeves MM (2013). Adults' past-day recall of sedentary time: reliability, validity, and responsiveness. Med Sci Sports Exerc.

[7] Washburn RA, Smith KW, Jette AM, Janney CA (1993). The physical-activity scale for the elderly (PASE)—development and evaluation. J Clin Epidemiol.

[8] Choe MA, Kim J, Jeon MY, Chae YR (2010). Evaluation of the Korean version of physical activity scale for the elderly (K-PASE). Korean J Women Health Nurs.

[9] Ngai SP, Cheung RT, Lam PL, Chiu JK, Fung EY (2012). Validation and reliability of the physical activity scale for the elderly in Chinese population. J Rehabil Med.

[10] Casartelli NC, Bolszak S, Impellizzeri FM, Maffiuletti NA (2015). Reproducibility and validity of the physical activity scale for the elderly (PASE) questionnaire in patients after total hip arthroplasty. Phys Ther.

[11] Tsang S, Royse CF, Terkawi AS (2017). Guidelines for developing, translating, andvalidatinga questionnaire in perioperative and pain medicine. Saudi J Anaesth.

[12] Roehrig B, Hoeffken K, Pientka L, Wedding U (2007). How many and which items of activities of daily living (ADL) and instrumental activities of daily living (IADL) are necessary for screening. Crit Rev Oncol/Hematol.

[13] Tanjani PT, Azadbakht M (2015). Psychometric properties of Persian version of the activities of daily living scale and instrumental activities of daily living scale in elderly. J Mazandaran Univ Med Sci.

[14] Wennie Huang WN, Perera S, Van Swearingen J, Studenski S (2010). Performance measures predict onset of activity of daily living difficulty in community-dwelling older adults. J Am Geriatr Soc.

[15] Podsiadlo D, Richardson S (1991). The timed "Up & Go": a test of basic functional mobility for frail elderly persons. J Am Geriatr Soc.

[16] Terwee CB, Mokkink LB, van Poppel MN, Chinapaw MJ, van Mechelen W, de Vet HC (2010). Qualitative attributes and measurement properties of physical activity questionnaires: a checklist. Sports Med.

[17] Vaughn K, Miller WC (2013). Validity and reliability of the Chinese translation of physical activity scale for the elderly (PASE). Disabil Rehabil.

[18] Alqarni AM, Vennu V, Alshamari SA, Bindawas SM (2018). Cross cultural adaptation and validation of the Arabic version of the physical activity scale for the elderly among community dwelling older adults in Saudi Arabia. Clin Interv Aging.

[19] Hagiwara A, Ito N, Sawai K, Kazuma K (2008). Validity and reliability of the physical activity scale for the elderly (PASE) in Japanese elderly people. Geriatr Gerontol Int.

[20] Shetty P (2012). Gray matter: aging in developing countries. Lancet.

[21] Schuit AJ, Schouten EG, Westerterp KR, Saris WH (1997). Validity of the physical activity scale for the elderly (PASE): according to energy expenditure assessed by the doubly labeled water method. J Clin Epidemiol.

[22] Salehi L, Taghdisi MH, Ghasemi H, Shokervash B (2010). To identify the facilitator and barrier factors of physical activity among elderly people in Tehran. Iran J Epidemiol.

[23] Washburn RA, McAuley E, Katula J, Mihalko SL, Boileau RA (1999). The physical-activity scale for the elderly (PASE)—evidence for validity. J Clin Epidemiol.

[24] Ku PW, Fox KR, Chen LJ, Chou P (2012). Physical activity and depressive symptoms in older adults: 11-year follow-up. Am J Prev Med.

[25] Salehi L, Eftekhar H, Mohammad K, Taghdisi MH, Shojaeizadeh D (2010). Physical activity among a sample of Iranians aged over 60 years: an application of the transtheoretical model. Arch Iran Med.

[26] Singh DKA, Rahman NNAA, Rajaratram BS, Yi TC, Shahar S (2018). Validity and reliability of physical activity scale for elderly in Malay language (PASE-M). Malays J Public Health Med.

[27] Ismail N, Hairi F, Choo WY, Hairi NN, Peramalah D, Bulgiba A (2015). The physical activity scale for the elderly (PASE): validity and reliability among community-dwelling older adults in Malaysia. Asia Pac J Public Health.

[28] Schrempft S, Jackowska M, Hamer M, Steptoe A (2019). Associations between social isolation, loneliness, and objective physical activity in older men and women. BMC Public Health.

[29] Dinger MK, Oman RE, Taylor EL, Vesely SK, Able J (2004). Stability and convergent validity of physical activity scale for the elderly (PASE). J Sports Med Phy Fit.

[30] Riebe D, Blissmer BJ, Greaney ML, Garber CE, Lees FD, Clark PG (2009). The relationship between obesity, physical activity, and physical function in older adults. J Aging Health.

[31] Chad KE, Reeden BA, Harrison EL, Ashworth NL, Shappared SM, Schutz SL, Bruner BG, Fisher KL, Lawson JA (2005). Profile of physical activity levels in community dwelling older adults. Med Sci Sports Exerc.

[32] Salehi L, Shokrvash B, Jamshidi E, Montazeri A (2014). Physical activity in Iranian older adults who experienced fall during the past 12 months. BMC Geriatr.

[33] Loland NW (2002). Reliability of the physical activity scale for the elderly (PASE). Eur J Sport Sci.

[34] Tavakol M, Dennick R (2011). Making sense of Cronbach’s alpha. Int J Med Educ.

