# Supplementary material for: Cultural adaptation and psychometric adequacy of the Persian version of the physical activity scale for the elderly (P-PASE)
Source: BMC Res Notes. 2019 Sep 2;12:555. doi: 10.1186/s13104-019-4591-7 (PMC6719368; doi:10.1186/s13104-019-4591-7)
Supplement: Supplementary file 1 — Additional file 1: Table S1. The mean of P-PASE components in the male and female. [file 13104_2019_4591_MOESM1_ESM.doc]

**TableS1: The mean of P-PASE components in the male and female**

|  | Male(n=101) | | | | Female(n=186) | | | P |
| --- | --- | --- | --- | --- | --- | --- | --- | --- |
| PASE Component | Sample mean | Weight | | Contribution to subtotal P-PASE Score | Sample mean | Weight | Contribution to subtotal P-PASE Score |
| Leisure time Activity(M±SD) | 27.47±17.12 | | | | 59.29±25.86 | | | <0.001 |
| Walking(h/day) | 1.17 | 20 | 23.40 | | 2.39 | 20 | 47.80 |
| Light Sport(h/day) | 0.07 | 21 | 1.47 | | 0.19 | 21 | 3.99 |
| Moderate (h/day) | 0.06 | 23 | 1.38 | | 0.11 | 23 | 2.53 |
| Strenuous Sport | 0.03 | 23 | 0.92 | | 0.19 | 23 | 4.37 |
| Muscular Strength/Endurance(h/day) | 0.01 | 30 | 0.3 | | 0.02 | 30 | 0.6 |
| House hold Activity(M±SD) | 57.17±25.88 | | | | 94.49±29.22 | | | <0.001 |
| Light Housework (%) | 81.20 | 25 | 20.30 | | 91.99 | 25 | 23.00 |
| Heavy Housework (%) | 78.22 | 25 | 19.56 | | 89.25 | 25 | 22.31 |
| Home Repair (%) | 6.93 | 30 | 2.08 | | 26.34 | 30 | 7.90 |
| Lawn Work/Yard Care (%) | 10.80 | 36 | 3.89 | | 47.31 | 36 | 17.03 |
| Caring for another Person (%) | 31.68 | 35 | 11.09 | | 69.29 | 35 | 24.25 |
| Work for pay or volunteer | 22.89±10.04 | | | | 25.2±11.08 | | | 0.13 |
| Job-Standing or walking(h/day) | 1.05 | 21 | 22.89 | | 1.20 | 21 | 26.88 |
| Total PASE Score(M±SD) | 106.69±25.7 | | | | 178.98±38.01 | | |  |
