# Supplementary material for: Cultural adaptation and psychometric adequacy of the Persian version of the physical activity scale for the elderly (P-PASE)
Source: BMC Res Notes. 2019 Sep 2;12:555. doi: 10.1186/s13104-019-4591-7 (PMC6719368; doi:10.1186/s13104-019-4591-7)
Supplement: Supplementary file 2 — Additional file 2. Persian version of PASE. [file 13104_2019_4591_MOESM2_ESM.doc]

**مقياس تعيين ميزان فعاليت جسمانی**

-در طي 7روز گذشته، معمولا"چه مدتي راصرف فعاليت هاي نشسته اي چون مطالعه کردن،تماشاي تلويزيون يا انجام کارهايي مانند انجام بافتني نموده اند:

هرگز(0) بندرت(1-2 روز) بعضي اوقات(3-4 روز) اغلب(5-7 روز)

-درصورتيکه پاره اي از وقت خودرا صرف اموري نشسته اي مانند مطالعه کردن، تماشاي تلويزيون ياانجام کارهايي نشسته اي مانند بافتني مي کرديد:

بطور متوسط چند ساعت در روز ر اصرف اين اعمال مي نموديد:

(0) کمتراز1 ساعت □

(1) کمتراز2 ساعت □

(2). 2تا 4 ساعت □

(3) بيش از4ساعت □

- در طي7روز گذشته ،معمولا"چقدر از وقتتان را صرف پياده روي در حياط يا بيرون خانه اتان به منظورتفريح، رفتن به محل کار بصورت پیاده ، رفتن پیاده به ديدن اقوام ودوستان يا رفتن به مسجد کرده اید:

هرگز(0) بندرت(1-2 روز) بعضي اوقات(3-4 روز) اغلب اوقات (5-7روز)

چه مقدار از وقت خود صرف کارهای فوق نموده اید :

(0) کمتر از1 ساعت □

(1) کمتر از2 ساعت □

(2). 2تا 4 ساعت □

(3) بيش از4ساعت □

- در طي7روز گذشته، معمولا" چقدر را صرف انجام ورزشهاي سبک و فعاليت هاي تفريحي مانند باغباني،چمن زني، رفتن خرید تفریحی، بازی با تخته نرد، بازی با شطرنج ،، نرمش سبک و فعاليت هايي ازاين نوع، نموده ايد

هرگز(0) بندرت (1-2 روز) بعضي اوقات (3-4 روز) اغلب اوقات (5-7روز)

بطور متوسط چند ساعت در روز را صرف اين اعمال مي نموديد:

(0) کمتراز1 ساعت □

(1) کمتر از2 ساعت □

(2). 2تا 4 ساعت □

(3) بيش از4 ساعت □

-در طي روز گذشته ،معمولا" چقدر را صرف انجام ورزش هايي با شدت متوسط و انجام فعاليت هاي تفريحي مانند شرکت در راه پیمایی های خانوادگی و یا دوستانه ، انجام بازی وسطی با اعضای خانواده و فعاليت هايي از اين نوع نموده ايد:

هرگز(0) بندرت (1-2 روز) بعضي اوقات (3-4 روز) اغلب اوقات (5-7روز)

بطور متوسط چند ساعت در روزرا صرف اين اعمال مي نموديد:

(0) کمتر از1 ساعت □

(1) کمتر از2 ساعت □

(2). 2تا 4 ساعت □

(3) بيش از4 ساعت □

در طي7روز گذشته، معمولا" چقدر را صرف انجام ورزش هايي شديد و يا انجام فعاليت هاي تفريحي شديدي مانند انجام شنا، کوه پیمایی و فعاليت هايي از اين نوع، نموده ايد:

هرگز(0) بندرت (1-2 روز) بعضي اوقات (3-4 روز) اغلب اوقات (5-7روز)

بطور متوسط چند ساعت در روز را صرف اين اعمال مي نموديد:

(0) کمتر از1 ساعت □

(1) کمتر از 2 ساعت □

(2). 2تا 4 ساعت □

(3) بيش از4ساعت □

در طي 7روز گذشته ،معمولا"چقدر را صرف انجام ورزش هاي خاصي براي تقويت عضلات يا افزايش تحمل(مانندبلند کردن وزنه) نموده ايد:

هرگز(0) بندرت (1-2 روز) بعضي اوقات (3-4 روز) اغلب اوقات (5-7روز)

بطور متوسط چند ساعت در روز را صرف اين اعمال مي نموديد:

- (0) کمتر از 1 ساعت □

(1) کمتر از 2 ساعت □

(2). 2تا 4 ساعت □

(3) بيش از 4 ساعت □

در طي 7روزگذشته،آيا زماني از وقت خود را صرف انجام فعاليت هاي خانگي سبکي مانند گردروبي ياشستن ظروف نموده ايد:

بلي□ خير□

در طي7روزگذشته، آيا شما فعاليت خانگي شديدي مانند جارو کردن، شستن کف زمين، شستن درب و پنجره ، شستن لباس ها با دست و یا اتو کردن لباس ها نموده ايد:

بلي□ خير□

آيا در طي 7روز گذشته، فعاليت هايي مانندفعاليت هاي زير راداشته ايد:

انجام تعمیرات مانند تعمیرات خانه و یا تعمیرات خودرو

بلي□ خير□

کارهايي مانند تمیز کردن حیاط، جمع کردن برگ درختان و مرتب نمودن باغچه و شستن خوددرو و غيره

بلي□ خير□

مراقبت از شخص ديگرمانند نوه هاو بچه هاي کوچک ياهر شخص وابسته برزگتر

بلي□ خير□

آيا در طي7 روزگذشته به سرکار رفته ايدياکارهاي داوطلبانه اي از اين نوع داشته ايد:

چند ساعت را صرف انجام کار و يا انجام امور داوطلبانه نموده ايد:

از دسته فعاليت هاي زير، کداميک توصيف مناسبي براي انجام فعاليت هايي است که شما بر سرکارتان يا انجام امور داوطلبانه انجام مي دهيد:

- دسته اول: اساسا"در برگيرنده فعاليتهاي نشسته ايي است که اندکي با حرکت دستها همراه باشد (مانند رانندگی، صلوات فرستادن با تسبیح، یا تایپ نمودن)

- شستن و راه متناوب (به عنوان مثال صندوقدار، کارگر ماشين آلات سبک، نگهبانی و...)

- راه رفتن متناوب باحمل بارهاي کم وزن مثل حمل میوه و سبزی خریداری شده از مغازه تا منزل

د- راه رفتن و داشتن کارهاي سنگين و يا حمل بارهاي سنگين ( مانند حمل وسایل منزل حین جابجایی منزل و اسباب کشی و یا خانه تکانی یا کارگری و حمل بار و یا کشاورزی)

متوسط زماني که صرف هر يک از اين فعاليت ها نموده ايد را به ساعت در روز مشخص نماييد ( در طي 7روز گذشته)
